# Clinical validation of the next-generation sequencing-based Extended RAS Panel assay using metastatic colorectal cancer patient samples from the phase 3 PRIME study

**DOI:** 10.1007/s00432-018-2688-3

**Published:** 2018-07-17

**Authors:** Nitin Udar, Catherine Lofton-Day, Jun Dong, Darcy Vavrek, A. Scott Jung, Kristen Meier, Anita Iyer, Ryan Slaughter, Karen Gutekunst, Bruce A. Bach, Marc Peeters, Jean-Yves Douillard

**Affiliations:** 1 Department of Clinical Genomics Assay Development, Oncology, Illumina, Inc., 5200 Illumina Way, San Diego, CA 92122 USA; 2 Department of Medical Sciences, Amgen Inc., Thousand Oaks, CA USA; 3 Department of Biostatistical Sciences, Amgen Inc., Thousand Oaks, CA USA; 4 Department of Biostatistics, Illumina, Inc., San Diego, CA USA; 5 Department of Research and Development, Amgen Inc., Thousand Oaks, CA USA; 6 Department of Clinical Affairs, Illumina, Inc., San Diego, CA USA; 7 Department of Global Oncology Development, Amgen Inc., Thousand Oaks, CA USA; 8 Department of Oncology, Antwerp University Hospital, Edegem, Belgium; 9 Department of Medical Oncology, ICO René Gauducheau, Nantes, France

**Keywords:** Gastrointestinal cancers, Colorectal, New software for data analysis, Mutation detection methods, Molecular diagnosis and prognosis

## Abstract

**Purpose:**

To validate a next-generation sequencing (NGS)-based companion diagnostic using the MiSeqDx^®^ sequencing instrument to simultaneously detect 56 *RAS* mutations in DNA extracted from formalin-fixed paraffin-embedded metastatic colorectal cancer (mCRC) tumor samples from the PRIME study. The test’s ability to identify patients with mCRC likely to benefit from panitumumab treatment was assessed.

**Methods:**

Samples from PRIME, which compared first-line panitumumab + FOLFOX4 with FOLFOX4, were processed according to predefined criteria using a multiplex assay that included input DNA qualification, library preparation, sequencing, and the bioinformatics reporting pipeline. NGS mutational analysis of *KRAS* and *NRAS* exons 2, 3, and 4 was performed and compared with Sanger sequencing.

**Results:**

In 441 samples, positive percent agreement of the Extended RAS Panel with Sanger sequencing was 98.7% and negative percent agreement was 97.6%. For clinical validation (*n* = 528), progression-free survival (PFS) and overall survival (OS) were compared between patients with *RAS* mutations (*RAS* Positive) and those without (*RAS* Negative). Panitumumab + FOLFOX4 improved PFS in *RAS* Negative patients (*P* = 0.02). Quantitative interaction testing indicated the treatment effect (measured by the hazard ratio of panitumumab + FOLFOX4 versus FOLFOX4) differed for *RAS* Negative versus *RAS* Positive for PFS (*P* = 0.0038) and OS (*P* = 0.0323).

**Conclusions:**

NGS allows for broad, rapid, highly specific analyses of genomic regions. These results support use of the Extended RAS Panel as a companion diagnostic for selecting patients for panitumumab, and utilization is consistent with recent clinical guidelines regarding mCRC *RAS* testing. Overall, approximately 13% more patients were detected with the Extended RAS Panel versus *KRAS* exon 2 alone.

**Clinical trial registry identifier:**

NCT00364013 (ClinicalTrials.gov).

**Electronic supplementary material:**

The online version of this article (10.1007/s00432-018-2688-3) contains supplementary material, which is available to authorized users.

## Introduction

Molecular genetic studies targeting metastatic colorectal cancer (mCRC) tumors have identified mutations in *KRAS* and *NRAS* that predict a lack of therapeutic response to epidermal growth factor receptor (EGFR) inhibitors (Bokemeyer et al. 2015; Douillard et al. 2013; Peeters et al. 2013; Van Cutsem et al. 2015). As a result, the European Society for Medical Oncology (ESMO) (Van Cutsem et al. 2016), the National Comprehensive Cancer Network (NCCN) (National Comprehensive Cancer Network 2017), and the American Society for Clinical Pathology, the College of American Pathologists, the Association for Molecular Pathology, and the American Society of Clinical Oncology recommended extended *RAS* mutation testing of *KRAS* and *NRAS* exon 2 (codons 12 and 13), exon 3 (codons 59 and 61), and exon 4 (codons 117 and 146) to improve patient selection for anti-EGFR treatment, thereby improving patient outcomes (Sepulveda et al. 2017).

PRIME (20050203) was a phase 3, multicenter, open-label, randomized trial that evaluated the treatment effect of panitumumab plus FOLFOX4 compared with FOLFOX4 alone as first-line therapy in 1183 patients with wild-type *KRAS* exon 2 mCRC (Douillard et al. 2010; Peeters et al. 2012). PRIME has been analyzed sequentially as additional information about activating *RAS* mutations has become available. Archived CRC tissue samples were most commonly isolated from the primary lesions of patients with mCRC. These samples were tested in a prospective–retrospective analysis for additional activating *RAS* mutations beyond *KRAS* exon 2 using Sanger sequencing. The ascertainment rate of tumor *RAS* status was 90% (*n* = 1060/1183). In-depth sequencing identified an additional 17% of samples that carried *RAS* mutations in addition to those originally identified in *KRAS* exon 2. These mutations predicted a lack of response to EGFR inhibition in patients who received panitumumab plus FOLFOX4. Moreover, patients treated with panitumumab plus FOLFOX4 with *RAS* mutations had worse progression-free survival (PFS; 7.3 months) and overall survival (OS; 15.5 months) compared with those who had no *RAS* mutations (PFS, 10.1 months; OS, 25.8 months) (Douillard et al. 2013).

Next-generation sequencing (NGS) has emerged as a powerful tool that provides broad, rapid, highly specific analyses of genomic regions of interest in a single assay (Shendure and Ji 2008). Because NGS has the capability to interrogate millions of DNA fragments in parallel, this technology decreases sequencing time, labor, and reagents, which significantly reduces cost and time to results compared with iterative single locus testing. The Extended RAS Panel is a United States Food and Drug Administration-approved, qualitative *in vitro* diagnostic NGS test developed for the simultaneous detection of 56 known activating mutations within exons 2, 3, and 4 of the *KRAS* and *NRAS* genes. The assay system includes sequence-specific reagents and consumables for DNA qualification, library preparation, and sequencing, as well as integrated data analysis and reporting software. The assay is intended for use with the MiSeqDx^®^ (Illumina, Inc., San Diego, CA, USA) sequencing instrument. The goal of this analysis was to clinically validate a companion diagnostic (CDx) test to aid in the identification of patients with mCRC eligible for treatment with panitumumab (Vectibix^®^, Amgen Inc., Thousand Oaks, CA, USA).

The clinical accuracy of the Extended RAS Panel was evaluated by comparing the results provided by the NGS assay to those of the reference method, Sanger bidirectional sequencing. For the clinical validation study, clinical outcomes were examined to determine if there was an improvement in PFS and OS in wild-type *RAS* patients treated with panitumumab plus FOLFOX4 versus FOLFOX4 alone when *RAS* status was determined by the Extended RAS Panel.

## Materials and methods

### Patients

Patient eligibility criteria for the randomized controlled PRIME study have been previously described (Douillard et al. 2010).

### Pathology assessment

All randomized patients with available and eligible formalin-fixed paraffin-embedded (FFPE) samples were evaluated by a board-certified pathologist at a Clinical Laboratory Improvement Amendments (CLIA)-certified laboratory. Microscopic inspection of hematoxylin and eosin stained slides was performed to ascertain the area of the tissue and the tumor. Only samples meeting a ≥ 50% tumor content were selected for DNA extraction. Samples containing < 50% tumor tissue were enriched using macrodissection to remove normal tissue content. For optimal yield of amplifiable DNA, the recommended cumulative tissue area was ≥ 240 mm^2^ (at least 8 × 5 µM serial sections). The results of the pathology review were recorded, and qualified samples were processed for DNA extraction by an external, independent CLIA-certified laboratory for *RAS* CDx testing and a second CLIA-certified laboratory for Sanger sequencing. Individuals involved in the *RAS* sample laboratory testing did not have access to treatment allocation or study clinical outcomes.

### Sample qualification, library preparation, and sequencing

**Fig. 1:**
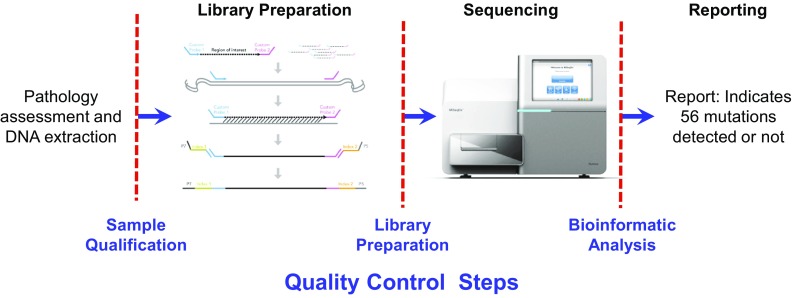
Extended RAS Panel assay workflow

The assay uses a dual strand approach using TruSeq Custom Amplicon (TSCA) technology (Illumina, Inc., San Diego, CA, USA) to distinguish true mutations from artifacts commonly found in DNA from FFPE tissue. Following DNA extraction using the QIAGEN FFPE extraction kit (QIAGEN, Germantown, MD, USA), the samples were prepared for NGS. The Extended RAS Panel assay involves three main steps (Fig. 1). The first step is to qualify the DNA sample to be used for the assay. Extracted DNA samples are subjected to quantitative polymerase chain reaction (qPCR), which assesses the sample’s quality and quantity, or amplifiability. The samples are qualified by measuring amplifiability relative to a control DNA template. The metric to assess the quality of FFPE DNA is the change in quantification threshold cycle (dCq) between sample and control DNA. If this dCq value is ≤ 5.0, the samples are eligible to advance to step two, library preparation. A dCq level of − 0.5 to 5.0 corresponds to approximately 1200 ng to 25 ng, respectively, of intact DNA used in the assay.

The second step is to prepare the samples for sequencing. Library preparation using TSCA consists of four key steps: hybridization, extension–ligation, PCR amplification, and library normalization. In hybridization, oligonucleotide probes targeting specific *RAS* mutations are hybridized to the sample genomic DNA (each DNA strand is independently targeted by two oligonucleotide probe pools). Extension–ligation connects the hybridized upstream and downstream oligonucleotides to form products that contain the *RAS*-specific oligonucleotides flanked by sequences required for amplification. PCR amplification amplifies the extension–ligation products using primers that add index sequences for multiplexing of numerous patient samples in a single assay, as well as common adapters required for cluster generation on the MiSeqDx sequencing instrument. Following library preparation, as a quality control (QC) measure, the samples are electrophoresed on an agarose gel to visualize the amplified product (ie, the library). At the end of this process, a PCR clean-up procedure purifies the PCR products (referred to as a library). Library normalization balances the quantity of each library to ensure equal library representation in the final pooled library. Following this step, the pooled library is loaded onto the MiSeqDx sequencing instrument for sequencing using sequencing by synthesis (SBS) chemistry. Up to 10 samples and two controls (positive and negative) can be sequenced together.

After sequencing is completed, the instrument software analyzes the data. During this analysis, sequencing reads can be traced back to their unique originating source sample based on their index sequence (ie, demultiplexing). Several quality parameters are assessed and a final report detailing whether any mutations are identified is generated.

The positive and negative controls are prepared in parallel with the samples and are included in every sequencing run. The positive control consists of two *RAS* panel mutations that have low allele frequency and is used to evaluate the performance of the library preparation and sequencing. The panel control must generate the expected genotype to be valid. If the control is invalid, processing errors may have occurred. The software will fail the entire sequencing run and all samples will appear as invalid. The negative control serves to detect issues like cross-contamination. If an unexpected outcome occurs in the negative control, the software will fail the entire sequencing run and all samples will appear as invalid.

Only results for the 56 mutations listed in Table S1 are reported. The panel mutations were selected based on four panitumumab clinical sequencing studies (Douillard et al. 2010; Patterson et al. 2013; Peeters et al. 2015; Schwartzberg et al. 2014), the Catalogue Of Somatic Mutations In Cancer (COSMIC) database, the NCCN guidelines (National Comprehensive Cancer Network 2017), and the Vaughn et al. publication (2011). For calculation of clinical accuracy, results from the Extended RAS Panel testing were compared with the output from the antecedent Sanger sequencing.

In the primary analysis of the PRIME study as reported by Douillard et al. (2010), the *therascreen* investigational use only PCR kit (QIAGEN) was used to identify *KRAS* exon 2 mutations by analyzing seven somatic mutations in *KRAS* codons 12 and 13 (ie, alleles G12A, G12D, G12R, G12C, G12S, G12V, or G13D). Tumor samples from patients identified as having one or more of the seven *KRAS* exon 2 mutations and with sufficient additional tissue sections that met previously described quality criteria underwent additional testing for mutations in exons 2, 3, and 4 of *KRAS* and *NRAS* using Sanger bidirectional sequencing. Samples identified to have no mutations in *KRAS* exon 2 as determined by the *therascreen* assay were also tested for other mutations in exon 2 of *KRAS*. These extended Sanger sequencing data, which included *KRAS* exons 3 and 4 and *NRAS* exons 2, 3, and 4 status, were compiled with Sanger data from Douillard et al. (2013) to provide complete exon coverage for the qualified PRIME samples (Douillard et al. 2013).

Both the Extended RAS Panel and Sanger sequencing provided qualitative assessments of mutation status for both *KRAS* and *NRAS*. After passing predefined quality parameters to meet sequencing validity requirements, the final result of each method was a binary qualitative assignment into either *RAS* Mutation Detected (*RAS* Positive) or *RAS* Mutation Not Detected (*RAS* Negative) based on the identification of any of the 56 panel mutations. For NGS, a *RAS* Positive result was assigned if at least one *RAS* panel mutation was detected, a *RAS* Negative result was assigned if no *RAS* panel mutations were detected, and an invalid result was assigned if the data from a sample or a sequencing run were of insufficient quality. For Sanger sequencing, a *RAS* Positive result was assigned if a *RAS* mutation was detected in at least one exon, a *RAS* Negative result was assigned if no *RAS* mutations were detected in any exons, and an invalid result was assigned if at least one exon had an invalid result and others were either wild-type or invalid.

After Extended RAS Panel testing was completed, the results (mutation detected or mutation not detected) were linked to the most recent clinical endpoints for PFS and OS (data cutoffs for PFS and OS were 2010 and 2013, respectively) from the PRIME study data.

### Statistical analysis

Accuracy was assessed by calculating the positive percent agreement (PPA) and negative percent agreement (NPA) between the Extended RAS Panel and Sanger sequencing utilizing the patient-level overall *RAS* results.

The goal of the clinical validation statistical analysis reported here was to demonstrate whether there was an improvement in PFS and OS in patients treated with panitumumab plus FOLFOX4 versus FOLFOX4 alone whose tumors were found to have none of the 56 *RAS* mutations as determined by the Extended RAS Panel (*RAS* Negative Analysis Set). All hypotheses tested and confidence intervals (CIs) described were two-sided unless otherwise stated. A 5% significance level was used to compare the treatment effect on PFS and OS in the *RAS* Negative Analysis Set in a sequential manner. Tests of PFS and OS on all efficacy analysis sets were considered descriptive. All *P* values were not corrected for multiplicity and were considered descriptive.

The efficacy analyses of PFS and OS focused on the most recent clinical endpoints, and disease assessment was based on blinded central review of imaging studies using modified Response Evaluation Criteria in Solid Tumors criteria. Time-to-event variables were summarized using hazard ratios (HRs), Kaplan–Meier (KM) curves, KM estimates for quartiles, and log-rank test *P* values. Point estimates with 95% CIs were calculated.

Quantitative interaction tests were performed to evaluate whether the treatment effect as measured by the HR (panitumumab plus FOLFOX4:FOLFOX4 alone) was the same in *RAS* Negative patients versus *RAS* Positive patients. Interaction was estimated as the ratio of the HR for the *RAS* Negative Analysis Set over the HR for the mutant *RAS* Positive Analysis Set using methods from Gail and Simon to determine the magnitude and direction of the effect size in the biomarker-identified populations (Gail and Simon 1985).

## Results

### Patients

Of 1183 randomized patients, 891 had valid Sanger sequencing results, 528 had valid NGS results with a dCq ≤ 5.0, and 441 had both Extended RAS Panel and Sanger results and were used for the accuracy analysis. Patients were not included for the following reasons: 358 (30.3%) had no available remaining tissue for *RAS* testing, 127 (10.7%) had insufficient tissue area, 13 (1.1%) had insufficient neoplastic area, 215 (18.2%) had DNA of insufficient quality (214 had a dCq > 5.0 and one had a nonestimable dCq value), 28 (2.4%) were QC failures (including gel check failures and sequencing QC invalids; samples with invalid gel results were repeated using a second library preparation), and one (0.08%) had sample processing errors. To determine whether there was bias introduced by the truncation in the number of samples available using both methods, baseline disease characteristics for the 441 patients were compared with those of the *RAS* Unevaluable Set (Table S2). There were no apparent differences between cases with available tissue and those without available tissue.

### Accuracy

Of the 441 patients with valid Extended RAS Panel and Sanger results, 211 (48%) were *RAS* Negative and 230 (52%) were *RAS* Positive by Sanger sequencing. Of the 230 *RAS* Positive patients, 227 were *RAS* Positive by the Extended RAS Panel [PPA = 98.7% (*n* = 227/230); 95% CI 96.2–99.7%]. Of the 211 *RAS* Negative patients by Sanger, 206 were *RAS* Negative by the Extended RAS Panel [NPA = 97.6% (*n* = 206/211); 95% CI 94.6–99.2%; Table 1]. The lower limit of the 2-sided 95% CI (exact Clopper–Pearson) for both PPA and NPA exceeded 90%, and therefore the acceptance criteria were met. Eleven patients had conflicting results between Sanger sequencing and the Extended RAS Panel analysis; however, only eight patients had discrepant patient-level results (Table S3).

**Table 1:**
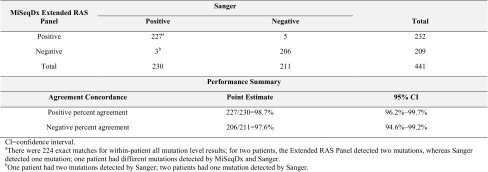
Positive and negative percent agreement of patient-level *RAS* results (dCq ≤ 5.0)

### Clinical validation

For patients whose tumors were *RAS* Negative, the median PFS time was 10.0 months for the panitumumab plus FOLFOX4 group and 9.2 months for the FOLFOX4 alone group. The HR (panitumumab plus FOLFOX4:FOLFOX4 alone) for PFS from a stratified Cox proportional hazards model was 0.700 (95% CI 0.516–0.948), indicating longer PFS for the panitumumab plus FOLFOX4 group than for the FOLFOX4 alone group. The stratified log-rank test revealed a statistically significant difference in PFS between the groups (*P* = 0.0206). For patients whose tumors were *RAS* Positive, the HR for PFS was 1.242 (95% CI 0.976–1.582), indicating longer PFS for the FOLFOX4 alone group than for the panitumumab plus FOLFOX4 group. Quantitative interaction testing indicated the treatment effect differed between patient groups, as measured by panitumumab plus FOLFOX4 versus FOLFOX4 alone, when comparing *RAS* Negative patients with *RAS* Positive patients (*P* = 0.0038; Table 2).

**Table 2:**
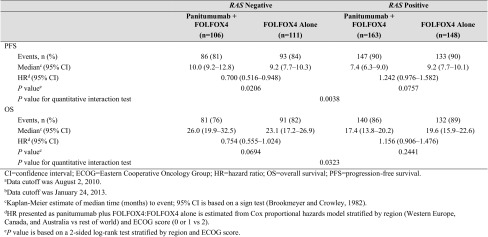
Primary efficacy parameters: PFS and OS for *RAS* Negative and *RAS* Positive

**Fig. 2:**
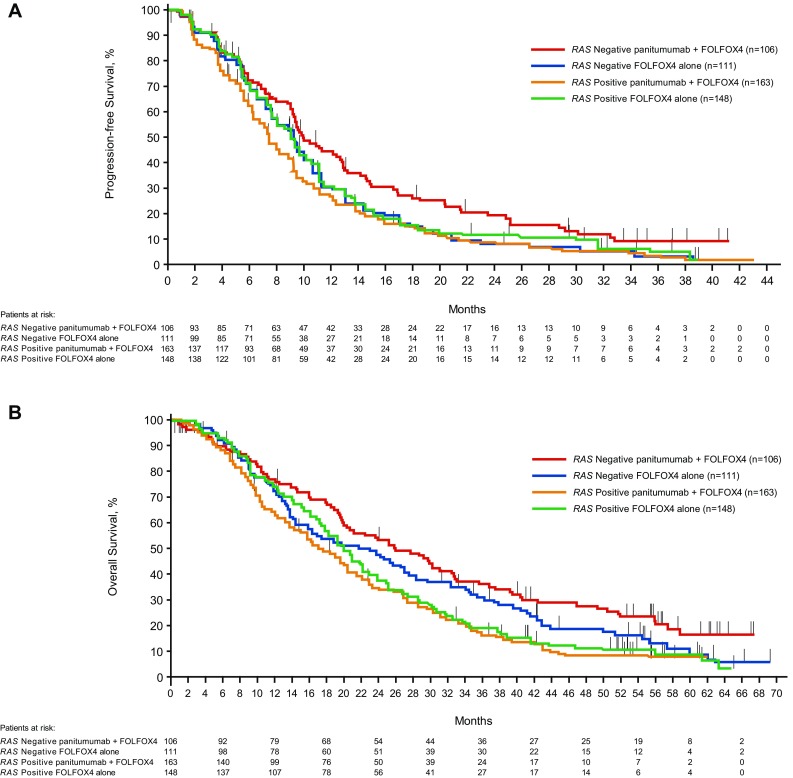
Kaplan–Meier plots of **A** progression-free survival^a^ and **B** overall survival^b^ according to *RAS* status and treatment. ^a^Data cutoff was August 2, 2010. ^b^Data cutoff was January 24, 2013

For *RAS* Negative patients, the median OS time was 26.0 months for the panitumumab plus FOLFOX4 group and 23.1 months for the FOLFOX4 alone group. The HR for OS from a stratified Cox proportional hazards model was 0.754 (95% CI 0.555–1.024; *P* = 0.0694), indicating longer survival for the panitumumab plus FOLFOX4 group than for the FOLFOX4 alone group, but it was not statistically significant. For *RAS* Positive patients, the HR was 1.156 (95% CI 0.906–1.476), indicating longer OS for the FOLFOX4 alone group than for the panitumumab plus FOLFOX4 group. Quantitative interaction testing revealed a statistically significant difference in OS (*P* = 0.0323; Table 2). Kaplan–Meier plots for PFS and OS in *RAS* Negative patients and *RAS* Positive patients are shown in Fig. 2a, b.

For the *KRAS* exon 2 Negative/*RAS* Positive subset (patients who did not have a mutation in *KRAS* exon 2, but did have a mutation in *KRAS* exons 3 or 4 or *NRAS* exons 2, 3, or 4), the trends were in the unfavorable direction: PFS and OS were longer in the FOLFOX4 alone group compared with the panitumumab plus FOLFOX4 group (Table 3).

**Table 3:**
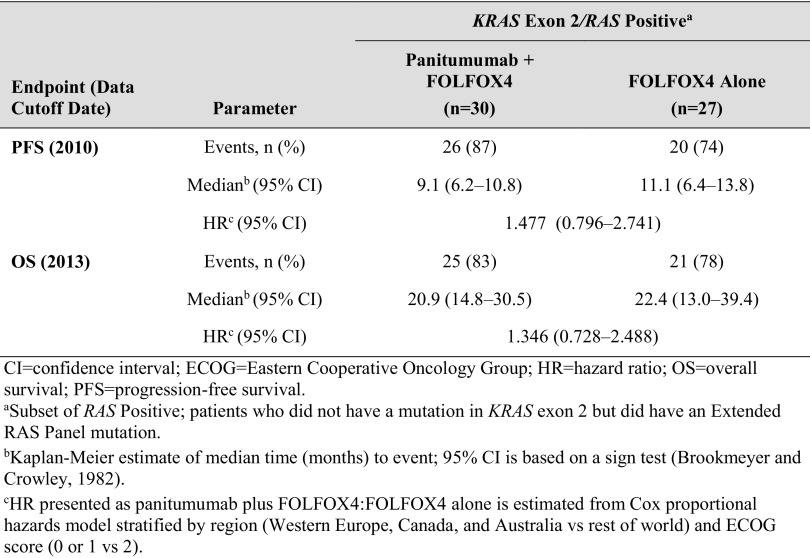
Primary efficacy parameters: PFS and OS for *RAS* Positive

The safety profile for panitumumab in patients with *RAS* Negative mCRC was similar to that in patients with *KRAS* exon 2 Negative mCRC.

### Mutation distributions

The overall prevalence of *RAS* mutations is shown in Table S4. Of all the mutations detected by the Extended RAS Panel (*n* = 311), mutations in *KRAS* exons 2, 3, and 4 occurred in 81.7, 4.2, and 6.1% of samples, respectively, and mutations in *NRAS* exons 2, 3, and 4 occurred in 2.6, 5.5, and 0% of detected mutations, respectively (Fig. S1, supplement).

### Mutation frequencies

**Fig. 3:**
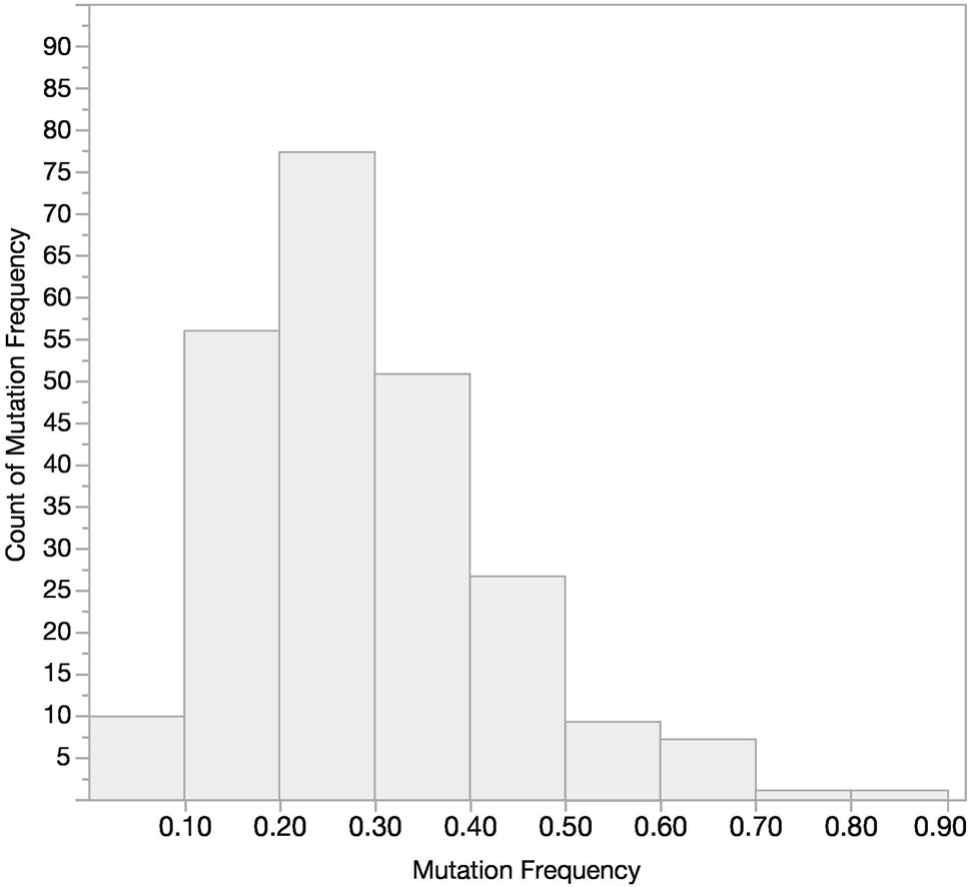
Extended RAS Panel mutation frequency distribution for positive samples (*n* = 235)

Using ≥ 50% tumor content in the FFPE specimens, the distribution of mutation frequencies observed in the PRIME study for samples that tested positive is shown in Fig. 3. The majority of specimens showed mutation frequencies in the 0.15–0.35 range. There were 14.9% of patients with a mutation frequency < 0.15, and 55.3% of patients with a mutation frequency ≥ 0.15 and ≤ 0.35.

## Discussion

A significant paradigm shift is occurring in cancer research and treatment approaches due to an increased focus on personalized medicine. As applied in mCRC therapy, the realized advantage is largely because of the ability to identify actionable driver mutations and match them to targeted therapies that can improve patient outcomes with potentially decreased exposure to toxicity (Amado et al. 2008; Moorcraft et al. 2013). One of the best known predictive biomarkers of a lack of therapeutic response is *KRAS* mutations in patients with mCRC treated with anti-EGFR antibodies. Historically, *KRAS* mutational analysis encompassed seven mutations in exon 2 (codons 12 and 13). Multiple clinical trials have subsequently identified additional activating *RAS* mutations that confer therapeutic resistance to anti-EGFR antibodies, and a meta-analysis demonstrated significantly shorter PFS and OS in patients with mutations in *KRAS* and *NRAS* (Therkildsen et al. 2014). Consequently, seminal clinical care guidelines for mCRC management agree that extended *RAS* testing should become standard of care (Allegra et al. 2016; National Comprehensive Cancer Network 2017; Sepulveda et al. 2017; Van Cutsem et al. 2016).

Next-generation sequencing technology has become widely available and has transformed the way genomic research is performed. Traditional testing techniques (eg, PCR, Sanger sequencing) are useful approaches but are limited in their ability to test for multiple types of genetic alterations and to screen multiple genes simultaneously. The CLIA laboratory that performed the Sanger sequencing for this study required a minimum of 350 ng of DNA. In contrast, NGS usually requires < 50 ng of DNA input and is optimized for FFPE samples, so it improves the diagnostic yield of biopsy samples that often contain limited amounts of cancerous tissue (Gagan and Van Allen 2015). These features have led to the development of the Extended RAS Panel, an NGS-based *in vitro* diagnostic test that analyzes 56 mutations in *KRAS* and *NRAS* in a single assay (Table S1). High depth of coverage, achieved by deep sequencing, increases detection of mutations at low frequency (below 10%). Multiplexing enables pooling of different patients’ DNA so they can be sequenced together in one single mixture, leading to improved laboratory throughput and further reducing time and costs. Multiplexing 56 targets also enables interrogation of all targets using a single DNA input and does not require individual reactions for each target. In addition, multiple mutations can be detected simultaneously. On completion of the sequencing run, the MiSeqDx sequencing instrument software analyzes the sequencing data, performs variant interpretation, and creates a final report, thus minimizing the demand for bioinformatics support and variations in the interpretation of results.

To assess clinical accuracy, results from the Extended RAS Panel were compared with Sanger sequencing in 441 patients who had been initially randomized to panitumumab plus FOLFOX4 or to FOLFOX4 alone in the PRIME trial (Douillard et al. 2010). Although Sanger sequencing is considered the “gold standard” technique, it does have several limitations. A study of *KRAS* mutation detection using pyrosequencing, melting curve analysis, and Sanger sequencing revealed that Sanger sequencing had a false positive rate of 11.1% (possibly caused by misattribution error or poor stringency amplification), a false negative rate of 6.1%, and a limit of detection of 15–20% (Tsiatis et al. 2010). In contrast, the National Cancer Institute-Molecular Analysis for Therapy Choice (NCI-MATCH) trial performed analytical validation of their NGS targeted assay covering 265 known somatic mutations and determined a sensitivity of 96.98%, a specificity of 99.99%, and a limit of detection (LOD) for single nucleotide variants of 2.8% (Lih et al. 2017). The Extended RAS Panel includes steps to interrogate both strands of DNA independently as well as provide high depth of coverage. This approach yields increased sensitivity and specificity with an LOD of 5% (Praxis™ Extended Panel 2017). This is particularly important when assessing rare mutations that exist in a heterogeneous population of neoplastic cells. In an evaluation of NPA, the NGS panel found mutations in five patients that were categorized as false positives because Sanger did not detect these mutations. We postulate that the NGS results represented true positives, while Sanger produced five false negatives. Nevertheless, concordance between the NGS assay results and Sanger results was excellent, with a PPA of 98.7% and an NPA of 97.6%.

Clinical validation of the Extended RAS Panel entailed an analysis of clinical outcomes from the PRIME study (Douillard et al. 2010). A lack of remaining mCRC tissue samples from the original cohort of 1183 randomized patients limited the number of cases for *RAS* mutational analysis by NGS. Ultimately, 528 samples passed QC measures to make up the evaluable *RAS* analysis set; 41% of samples were categorized as *RAS* Negative and 59% as *RAS* Positive. This is consistent with the Douillard et al. (2013) findings of 48% *RAS* Negative and 52% *RAS* Positive (Douillard et al. 2013). The comparison of the nonparametric Cox proportional hazards model of PFS between *RAS* Negative patients receiving panitumumab plus FOLFOX4 versus FOLFOX4 alone (*n* = 217) revealed a statistically significant difference of *P* = 0.02 (log-rank test) with an HR of 0.700 (95% CI 0.516–0.948). In the *RAS* Positive cohort, median PFS was 7.4 months with panitumumab plus FOLFOX4 and 9.2 months with FOLFOX4 alone (HR 1.24; 95% CI 0.976–1.582), suggesting a lack of clinical benefit with panitumumab, and potential detrimental effect of treatment, in this population. Quantitative interaction testing provided further support of the difference in PFS outcomes between *RAS* Positive and *RAS* Negative groups (*P* = 0.0038). In addition, the NGS assay produced HRs for PFS that were nearly identical to the PRIME reanalysis using Sanger sequencing with *RAS* Negative and *RAS* Positive HRs of 0.72 and 1.31, respectively (Douillard et al. 2013).

Evaluation of median OS revealed that *RAS* Negative patients treated with panitumumab plus FOLFOX4 lived longer (median, 26.0 months) than those treated with FOLFOX4 alone (23.1 months). The associated HR was 0.754 (95% CI 0.555–1.024; *P* = 0.07). In the *RAS* Positive group, median OS was 17.4 months in the panitumumab plus FOLFOX4 arm compared with 19.6 months with FOLFOX4 alone. Although the HR of 1.156 (95% CI 0.906–1.476) was not statistically significant, the differences observed in favor of chemotherapy alone are aligned with the findings of two large meta-analyses (Sorich et al. 2015; Therkildsen et al. 2014). As with PFS, quantitative interaction analysis confirmed that panitumumab treatment affected *RAS* Negative and *RAS* Positive patients differently, with a *P* value of 0.0323. Moreover, the PRIME study reported very similar HRs for OS in *RAS* Negative patients (median OS, 25.8 months versus 20.2 months; HR = 0.77; *P* = 0.009) and the *RAS* Positive group (median OS, 15.5 months versus 18.7 months; HR =  1.21; *P* = 0.001) (Douillard et al. 2013). The lack of statistical significance reported here is likely due to the small sample size in this study (*n* = 441 versus *n* = 1060 for the primary analysis), which likely resulted from the lack of remaining samples available and insufficient DNA quality. The low prevalence of individual mutations in *KRAS* exons 3 and 4 and *NRAS* exons 2, 3, and 4 range from 0.5 to 6.7% (Sorich et al. 2015) and help to retain the balance between the *RAS* Negative and *RAS* Positive Analysis Sets.

The distribution of mutations found within the *KRAS* and *NRAS* exons in this study are similar to those reported in published data (Sorich et al. 2015). Additionally, the distribution of mutation frequencies was also within the expected range given minimum 50% tumor content.

This analysis compared *RAS* testing results from Sanger sequencing and from the Extended RAS Panel for patients with mCRC treated with panitumumab. Utilization of this assay is consistent with recent clinical care guidelines regarding *RAS* testing in mCRC (Sepulveda et al. 2017). Overall, NGS allows for broad, rapid, highly specific analysis of genomic regions of interest, and these results support the use of the Extended RAS Panel as a companion diagnostic for the selection of patients who may derive benefit from panitumumab therapy. Testing for *KRAS* and *NRAS* mutations is recommended by both the NCCN and ESMO guidelines for all patients with mCRC; only patients with wild-type *RAS* tumors are eligible for anti-EGFR therapy (National Comprehensive Cancer Network 2017; Van Cutsem et al. 2016).

## Electronic supplementary material

Below is the link to the electronic supplementary material.

Supplementary material 1 (PDF 157 KB)
