# Supplementary material for: Clinical validation of the next-generation sequencing-based Extended RAS Panel assay using metastatic colorectal cancer patient samples from the phase 3 PRIME study
Source: J Cancer Res Clin Oncol. 2018 Jul 17;144(10):2001–10. doi: 10.1007/s00432-018-2688-3 (PMC6153611; doi:10.1007/s00432-018-2688-3)
Supplement: Supplementary file 1 — Supplementary material 1 (PDF 157 KB) [file 432_2018_2688_MOESM1_ESM.pdf]

Clinical validation of the next-generation sequencing–based Extended RAS Panel assay using metastatic colorectal cancer patient samples from the phase 3 PRIME study

Journal of Cancer Research and Clinical Oncology

Nitin Udar, Catherine Lofton-Day, Jun Dong, Darcy Vavrek, A. Scott Jung, Kristen Meier, Anita Iyer, Ryan Slaughter, Karen Gutekunst, Bruce A. Bach, Marc Peeters, and Jean-Yves Douillard

Corresponding author: Nitin Udar, PhD
Illumina, Inc.
5200 Illumina Way
San Diego, CA 92122
Phone: 1-858-699-6416
Email: greatbioinformatics@yahoo.com

Supplementary Material

Table S1. Extended RAS Panel Summary of 56 Mutations

Gene and Exon	Amino Acid	Mutation	Location
KRAS Exon 2	p.Gly12Ser	G>A	c.34
	p.Gly12Arg	G>C	c.34
	p.Gly12Cys	G>T	c.34
	p.Gly12Asn	GG>AA	c.34_35
	p.Gly12Phe	GG>TT	c.34_35
	p.Gly12Trp	GGT>TGG	c.34_36
	p.Gly12Asp	G>A	c.35
	p.Gly12Ala	G>C	c.35
	p.Gly12Val	G>T	c.35
	p.Gly13Arg	G>C	c.37
	p.Gly13Cys	G>T	c.37
	p.Gly13Asp	G>A	c.38
	p.Gly13Glu	GC>AA	c.38_39
	p.Gly13Asp	GC>AT	c.38_39
	p.Gly13Val	GC>TT	c.38_39
KRAS Exon 3	p.Ala59Thr	G>A	c.175
	p.Ala59Gly	C>G	c.176
	p.Gln61Lys	C>A	c.181
	p.Gln61Glu	C>G	c.181
	p.Gln61Arg	A>G	c.182
	p.Gln61Leu	A>T	c.182
	p.Gln61His	A>C	c.183
	p.Gln61His	A>T	c.183
KRAS Exon 4	p.Lys117Asn	A>C	c.351
	p.Lys117Asn	A>T	c.351
	p.Ala146Thr	G>A	c.436
	p.Ala146Pro	G>C	c.436
	p.Ala146Val	C>T	c.437
NRAS Exon 2	p.Gly12Ser	G>A	c.34
	p.Gly12Arg	G>C	c.34
	p.Gly12Cys	G>T	c.34
	p.Gly12Asn	GG>AA	c.34_35
	p.Gly12Phe	GG>TT	c.34_35
	p.Gly12Trp	GGT>TGG	c.34_36
	p.Gly12Asp	G>A	c.35
	p.Gly12Ala	G>C	c.35
	p.Gly12Val	G>T	c.35
	p.Gly13Arg	G>C	c.37
	p.Gly13Cys	G>T	c.37
	p.Gly13Asp	G>A	c.38

	p.Gly13Val	G>T	c.38
	p.Gly13Glu	GT>AA	c.38_39
	p.Gly13Glu	GT>AG	c.38_39
NRAS Exon 3	p.Ala59Thr	G>A	c.175
	p.Ala59Gly	C>G	c.176
	p.Gln61Lys	C>A	c.181
	p.Gln61Glu	C>G	c.181
	p.Gln61Arg	A>G	c.182
	p.Gln61Leu	A>T	c.182
	p.Gln61His	A>C	c.183
	p.Gln61His	A>T	c.183
NRAS Exon 4	p.Lys117Asn	G>C	c.351
	p.Lys117Asn	G>T	c.351
	p.Ala146Thr	G>A	c.436
	p.Ala146Pro	G>C	c.436
	p.Ala146Val	C>T	c.437

Table S2. Baseline Disease Characteristics for Efficacy Analysis Sets[a]

Characteristic[b]	*RAS* Negative: Panitumumab + FOLFOX4 (n=106)	*RAS* Negative: FOLFOX4 Alone (n=111)	*RAS* Positive: Panitumumab + FOLFOX4 (n=163)	*RAS* Positive: FOLFOX4 Alone (n=148)	Unevaluable *RAS*: Panitumumab + FOLFOX4 (n=324)	Unevaluable *RAS*: FOLFOX4 Alone (n=331)
Primary tumor type, n (%)						
Colon cancer	78 (74)	72 (65)	117 (72)	115 (78)	199 (61)	211 (64)
Rectal cancer	28 (26)	39 (35)	46 (28)	33 (22)	125 (39)	120 (36)
Adenocarcinoma differential of primary tumor well or moderately differentiated, n (%)	71 (67)	70 (63)	123 (76)	109 (74)	219 (68)	223 (67)
Months since primary diagnosis,[c] mean (SD)	10.0 (18.1)	7.7 (14.3)	8.7 (15.4)	7.4 (15.0)	9.4 (20.3)	10.4 (18.4)
Months since metastatic disease diagnosis,[c] mean (SD)	2.8 (4.8)	2.1 (1.9)	2.1 (2.1)	2.6 (6.5)	2.1 (4.7)	2.2 (3.8)
≥3 sites of metastatic disease, n (%)	39 (37)	47 (42)	74 (45)	66 (45)	166 (51)	150 (45)
Location of sites of metastatic disease, n (%)						
Liver only	17 (16)	22 (20)	25 (15)	21 (14)	52 (16)	54 (16)
Liver plus other sites	75 (71)	74 (67)	113 (69)	111 (75)	231 (71)	230 (69)
ECOG performance status, n (%)						
0 – Asymptomatic	62 (58)	56 (50)	86 (53)	76 (51)	184 (57)	188 (57)
1 – Symptomatic, ambulatory	37 (35)	48 (43)	70 (43)	66 (45)	123 (38)	124 (37)
2 – Symptomatic, >50% out of bed	7 (7)	6 (5)	7 (4)	6 (4)	17 (5)	17 (5)
Elevated CEA above normal range, n (%)	78 (75)	85 (80)	138 (87)	119 (83)	262 (83)	254 (80)
Baseline LDH concentration, n (%)						
≥1.5 × ULN	24 (23)	29 (26)	51 (31)	41 (28)	105 (32)	107 (32)
≥2.0 × ULN	17 (16)	21 (19)	35 (21)	29 (20)	74 (23)	72 (22)
Alkaline phosphatase ≥2.0 × ULN, n (%)	19 (18)	23 (21)	41 (25)	35 (24)	92 (28)	70 (21)

CEA=carcinoembryonic antigen; ECOG=Eastern Cooperative Oncology Group; LDH=serum lactate dehydrogenase; ULN=upper limit of normal.
[a]The data cutoff date for this analysis was August 28, 2009.
[b]Percentages are based on the number of patients randomized.
[c]Date of randomization minus date of primary diagnosis or metastatic disease diagnosis.

Table S3. Listing of Discrepant Calls (FP and FN) at the Mutation Level (dCq ≤5.0)

Sanger Result	Extended RAS Panel Result	Disagreement Location	
		FN	FP
KRAS 3 c.181C>A	Mutation negative	*KRAS* 3 c.181C>A	•
KRAS 2 c.35G>T	Mutation negative	*KRAS* 2 c.35G>T	•
KRAS 2 c.34G>A, *KRAS* 2 c.35G>T	Mutation negative	*KRAS* 2 c.34G>A, *KRAS* 2 c.35G>T	•
Mutation negative	*KRAS* 2 c.35G>A	•	*KRAS* 2 c.35G>A
Mutation negative	*KRAS* 2 c.35G>C	•	*KRAS* 2 c.35G>C
Mutation negative	*KRAS* 2 c.35G>T	•	*KRAS* 2 c.35G>T
Mutation negative	*NRAS* 3 c.181C>A	•	*NRAS* 3 c.181C>A
Mutation negative	*NRAS* 2 c.34G>T	•	*NRAS* 2 c.34G>T
KRAS 4 c.351A>C	*NRAS* 2 c.38G>A	*KRAS* 4 c.351A>C	*NRAS* 2 c.38G>A
KRAS 2 c.34G>T	*KRAS* 2 c.34G > T, *KRAS* 2 c.35G>C	•	*KRAS* 2 c.35G>C
KRAS 2 c.38G>A	*KRAS* 2 c.35G>T, *KRAS* 2 c.38G>A	•	*KRAS* 2 c.35G>T

Table S4. Prevalence of *RAS* Mutations

All *RAS* Wild-Type	Any *RAS* Mutant	
47.39%	52.61%	
**All *RAS* Wild-Type**	***KRAS* Exon 2 Mutant**	***KRAS* Exon 2 Wild-Type, Mutant *RAS***
47.39%	39.82%	12.78%

Fig S1. Distribution of detected *KRAS* and *NRAS* exons

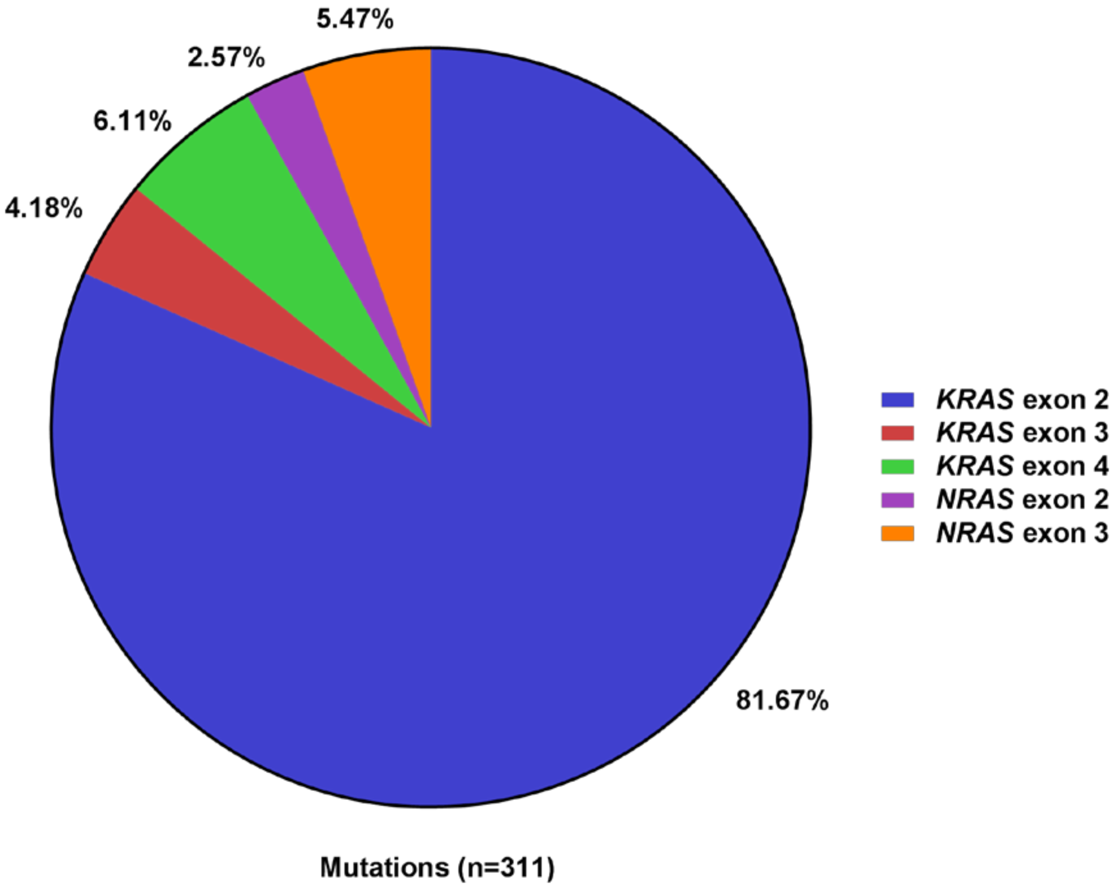

Figure generated using GraphPad Prism 7.
